# Inhibition of Major Virulence Pathways of *Streptococcus mutans* by Quercitrin and Deoxynojirimycin: A Synergistic Approach of Infection Control

**DOI:** 10.1371/journal.pone.0091736

**Published:** 2014-03-12

**Authors:** Sadaf Hasan, Kunal Singh, Mohd Danisuddin, Praveen K. Verma, Asad U. Khan

**Affiliations:** 1 Medical Microbiology and Molecular Biology Lab, Interdisciplinary Biotechnology Unit, Aligarh Muslim University, Aligarh, India; 2 National Institute of Plant Genome Research, Aruna Asaf Ali Marg, New Delhi, India; The Scripps Research Institute and Sorrento Therapeutics, Inc., United States of America

## Abstract

**Objectives:**

To evaluate the synergistic effect of Quercitrin and Deoxynojirimycin (DNJ) together with their individual inhibitory effect against virulence pathways of *Streptococcus mutans*.

**Methodology:**

MICs of both the compounds were determined by the microdilution method, followed by their *in vitro*synergy using checkerboard and time kill assay. The nature of interaction was classified as synergistic on the basis of fractional inhibitory concentration index (FICI) value of ≤0.5. Furthermore, the activity of Quercitrin and DNJ was evaluated individually and in combination against various cariogenic properties of *S. mutans* UA159 such as acidogenesis, aciduracity, glucan production, hydrophobicity, biofilm and adherence. Moreover, expression of virulent genes in *S. mutans* was analysed by quantitative RT- PCR (qRT-PCR) and inhibition of F_1_F_0_-ATPase, lactate dehydrogenase and enolase was also evaluated. Finally, scanning electron microscopy (SEM) was used to investigate structural obliteration of biofilm.

**Results:**

The *in vitro* synergism between Quercitrin and DNJ was observed, with a FICI of 0.313. Their MIC values were found to be 64 μg/ml and 16 μg/ml respectively. The synergistic combination consistently showed best activity against all the virulence factors as compared to Quercitrin and DNJ individually. A reduction in glucan synthesis and biofilm formation was observed at different phases of growth. The qRT-PCR revealed significant downregulation of various virulent genes. Electron micrographs depicted the obliteration of biofilm as compared to control and the activity of cariogenic enzymes was also inhibited.

**Conclusions:**

The whole study reflects a prospective role of Quercitrin and DNJ in combination as a potent anticariogenic agent against *S. mutans*.

## Introduction

Dental caries is a multifactorial infection, is characterized by progressive destruction of tooth enamel [1].*S. mutans*, a member of the oral micro flora, is considered to be the primary causative agent of dental caries (or tooth decay) and is one of the best known biofilm forming bacterium [2]. It has also been reported that *S. mutans* adhere to damaged cardiac tissues which is marked as a significant event in the pathogenesis of chronic infective endocarditis [3], with a death rate of up to 50% in spite of antibiotic treatments [4]. The aetiology of dental caries is associated with (i) bacterial fermentation of dietary carbohydrates resulting in acid production (ii) its ability to produce glucosyltransferases (GTFs), which leads to the synthesis of intracellular polysaccharides (IPS) and extracellular polysaccharides (EPS) and (iii) its attachment to the tooth pellicle mediated by glucans [5], [6]. The combination of these characteristic mechanisms acquired by *S. mutans* aid its effective colonization in the oral cavity and regulate the transformation from non-pathogenic to extremely cariogenic dental plaque biofilms [7]. Other fundamental cariogenic properties of *S. mutans* include the membrane-bound F_1_F_0_-ATPase system, lactate dehydrogenase and enolase. The membrane bound F_1_F_0_-ATPasesystem protects *S. mutans* against environmental acid stress by regulating pH homeostasis. This attribute determines the aciduracity or acid tolerance capability in *S. mutans* [8]. Lactate dehydrogenase (LDH) responsible for producing lactic acid and enolase (a glycolytic enzyme) produces phosphoenolpyruvate (PEP), which is a key component of the PEP:carbohydrate phosphotransferase system (PTS), further contributes to the promotion of cariogenecity by *S. mutans* [9].Several studies have shown that mutants of *S. mutans* which lack aforementioned virulence factors, are less cariogenic being more susceptible to different environmental stresses as compared to the wild strain [10], [11].There is an abundance of Indian medicinal herbs that are employed for the treatment of dental caries as they bear low or no toxicity, albeit the pure compounds have mostly reported to show better activity than the crude extracts. Supportingly, a study demonstrated that purified compound of *M. alba* showed an 8-fold greater reduction of MIC against *S. mutans* than the crude extract [12]. The quest for plants with medicinal properties will continue to receive attention but taking into consideration the recent emergence of microbes which are unaffected by most of the antimicrobial drugs and posing a challenge for the treatment of infections [13], there is an urgent need to come up with new antimicrobials which are less toxic and more efficient in combating such microorganisms. Another effective approach is described by combinational therapy which has been proved to be an effective alternative to monotherapy [14], [15]. Moreover, studies have demonstrated that those compounds which failed initially as antimicrobials, drastically enhanced the effectiveness ofother chemotherapeutic agent [16].Therefore, we have conducted this study using purified plant based compounds Quercitrin and Deoxynojirimycin (DNJ) in order to meet the need of efficiency with low toxicity levels [12], [17].Earlier studies have reported the significance of Quercitrin and DNJ in medicine. Quercitrin (quercetin 3- O-alpha- L-rhamnopyranoside), a flavonoid glycoside has been reported by many researchers for its wide array of pharmacological activities such as, anti-inflammatory [18], antileishmanial [19], antimelanogenic [20],prevention oflipid peroxidation [21] and protectiveagainst UVB-induced oxidative damage of skin [22].However, its activity against cariogenic properties of *S. mutans* has not been explored.Moreover, DNJis known to prevent diet-induced obesity [23], hepatitis C virus [24], modulate glucose metabolism and has anti-diabetic effects [25].DNJ, nevertheless, is known for its anti-biofilm effect but its activity and mechanism of action as pure compound is not clear [12].

Hence, in the present background we have initiated our study to explore the potential of Quercitrin and Deoxynojirimycin (DNJ) alone as well as in combination with each other (synergistically) against various virulence attributes of *S. mutans*. Their mechanisms of action on aforementioned bacterial virulence factors were also elucidated.

## Material and Methods

### Ethics statement

In this study self- samples of saliva and blood were taken for research purpose only. The blood was taken by the technically expert person. This research methodology was conducted in accordance with institutional ethical standards. The blood samples were taken from the person who has performed experiments (Ms. Sadaf Hasan). The samples were taken after her consent as she personally agreed to take her samples and gave consent verbally, since this is an author's own study who has used her samples. The verbal consent was documented in form of certificate from the chairman of ethical committee and this study has been specifically approved by the institutional ethical committee (IEC) under an approval number R. No. 1960/VC dated 03/6/13.

### Test bacterium and chemicals

The bacterial strain used in this study was *S. mutans*(UA159) (Institute of Microbial Technology, Chandigarh, India), which was grown in Brain Heart Infusion (BHI) Broth (Himedia Labs, Mumbai, India) at 37 °C. All chemicals and compounds unless otherwise stated, were purchased from Sigma-Aldrich Corp. (St. Louis, MO, U.S.A).1- Deoxynojirimycin (C_6_H_13_NO_4_) has a molecular weight of 163.17 and Quercitrin (C_21_H_20_O_11_) has a molecular weight of 448.38. (The purity of compoundswas 99%). The polyclonal antibodyagainst Ag I/II was a generous gift by Prof.M.M. Russell, State University of New York, U.S.A.

### Determination of Minimum inhibitory concentration (MIC) and bactericidal concentration (MBC)

The MIC and MBC of the compounds against *S. mutans* was determined by double dilution method as described previously [1]. The MIC was determined as the lowest concentration totally inhibiting the visible bacterial growth after 24 h of incubation at 37 °C. However, MBC was determined by subculturing the test dilutions on a tryptic soya agar plates, incubated further for 24 h. The highest dilution that resulted in no bacterial growth on solid medium was taken as MBC. All these determinations represent the mean of three independent experiments.

### Checkerboard microdilution assay

The *in vitro* interaction between Quercitrin and Deoxynojirimycin (DNJ) was evaluated by a two dimensional microdilution assay using 96 wells microtitre plate [14].To assess the effect of the combination to be synergistic, indifferent or antagonistic FICI (fractional inhibitory concentration index) was calculated. The following formulas were used to calculate the FICI of a combination.

FIC of compound A  =  (MIC of compound A in combination)/(MIC of compound A alone).

FIC of compound B  =  (MIC of compound B in combination)/(MIC of compound B alone).

FICI  =  FIC of compound A + FIC of compound B.

Synergy was defined as an FIC index value of ≤0.5. Indifference or absence of interaction was defined as FICI value >0.5 but <4. Antagonism was defined as an FICI of >4 [14].

### Time kill assay

Time-kill assay was performed according to the method as described earlier with slight modifications [15].Briefly, an exponentially growing culture of *S. mutans* was diluted to 1× 10^7^ CFU/ml in BHI medium for inoculation. Tubes containing BHI, bacterial species and the test concentrations of compounds [(i.e., sub- MIC levels of Quercitrin (32 μg/ml), Deoxynojirimycin (DNJ) (8 μg/ml) & their combination Q+D (32 μg/ml of Quercitrin+8 μg/ml of DNJ)] and the vehicle control (containing BHI medium, bacterial species and 1% DMSO) were incubated at 37 °C with continuous shaking. Samples were obtained at pre-determined time points (0, 2, 4, 8, 12 and 24 h after incubation). At each sample time a 500 μL aliquot was withdrawn from each tube and 10 fold dilutions were prepared in 0.85% saline. A 50 μL of the diluted sample was then plated onto BHI agar plates. These plates were incubated at 37 °C for 24 h, and colony counts were determined. Synergy was defined as a ≥100-fold or 2-log_10_ reduction in colony count at 24 h of incubation by the combination compared to single agent/compound showing maximum activity and as a ≥100-fold decrease in colony count compared with the starting inoculum. Contrastingly, indifference (or additivity) was defined as a <10-fold change in colony count at 24 h of incubation by the combination compared with the most active agent/compound alone. Antagonism was defined as a ≥100-fold increment in colony count at 24 h of incubation by the combination compared with that by the most active agent/compound alone [14].

### Glycolytic pH drop assay

The degree of the glycolytic pH drop by *S. mutans*cells was measured as described earlier with slight modification [26].Briefly, *S. mutans*cells were harvestedfrom the suspension culture (OD_600_ = 0.5). A salt solution (1 mM MgCl_2_+50 mM KCl) was used to wash these cells twice, prior to suspending them in the same solution containing the test concentration of the compounds (sub- MIC levels of Quercitrin, DNJ and Q+D) or the vehicle control (final concentration: 1% DMSO). The pH was set between 7.2–7.4 with 0.2 M KOH solution. Adequate glucose was then added to obtain a concentration of 1% (w/v) and the decrease in pH was evaluated over a period of 60 min using a glass electrode. The initial rate of the pH drop was taken as pH values between 0–10 min, which contributes to give the best measure of the acidogenesis capacity of the cells. This experiment was conducted independently for Quercitrin, DNJ and Q+D. All these experiments were performed in triplicates.

### F_1_F_0_-ATPase activity assay

The F_1_F_0_-ATPase activity was determinedusing permeabilized cells of *S. mutans*by adding the cells to 10% toluene (v/v) followed by series of freezing and thawing as described elsewhere [27]. The F_1_F_0_-ATPase activity was evaluated in terms of the release of inorganic phosphate in the following reaction mixture: 75 mmol of Tris-maleate buffer (pH 7.0) containing 5 mM adenosine -5′ -triphosphate (ATP), 10 mmol of MgCl_2_, permeabilized cells, and test concentration of the compound (sub- MIC levels of Quercitrin, DNJ and their combination Q+D) or the vehicle control (final concentration: 1% DMSO). The released phosphate was determined as previously described [28].The results were expressed as enzymatic activity as compared to control (untreated).

### Lactate dehydrogenase assay

To determine the Lactate dehydrogenase (LDH) activity,*S. mutans*cells were collected at late exponential phase (OD_600_ = 0.8)and incubated at 37°C with Tris-HCl buffer, pH 7.0; containing 0.5 mg/ml of lysozyme for 1 h. This lysate was then sonicated on ice consisting 2 cycles of 60 s each and the cell-free supernatant was collected by centrifugation for 10 min, 14000×g at 4°C. Furthermore, the crude extract was dialyzed at 4°C overnight against 10 mM phosphate buffer having a pH 6.9. The dialyzed formulation was defined as crude LDH. Its entire protein content was measured by the Bradford method [29].

In order to determine the activity of LDH, the crude LDH was pretreated with sub- MIC level of the compounds at room temperature for 30 min, and the LDH activity was estimated by measuring the rate of nicotinamide adenine dinucleotide (NADH) oxidation at 340 nm [30]. The standard reaction mixture (in a total volume of 200 μl) contained: 180 μl of 50 mM phosphate-buffered saline pH 6.9 with 0.167 mM NADH and 10 mM sodium pyruvate; 10 μl of 1 mM FDP (fructose 1, 6-diphosphate) and 10 μl of pretreated LDH. Results were expressed as enzymatic activity compared to that of the control. This experiment was conducted independently for Quercitrin, Deoxynojirimycin (DNJ) and the combination Q+D. All these experiments were performed in triplicates.

### Enolase activity assay

To evaluate the Enolase activity permeabilized cells of *S. mutans*were used by a method described previously [27].Permeabilized cells were pretreated with sub- MIC levels of the compounds at room temperature for 30 min. The enolase activity was directly monitored by the formation of phosphoenolpyruvate (PEP) at 240 nm. The standard reaction mixture (in a total volume of 200 μl) contained 180 μl of 20 mM KPO_4_ buffer, pH 6.5 with 2 mM MgSO_4_; 10 μl of 17.6 mM D- (+)- 2-phosphoglycerate and 10 μl of permeabilized cells already treated with the test concentration of the compounds. Results were expressed as enzymatic activity relative to that of the untreated control [9].

### GTF preparation, its activity and glucan synthesis assay

The crude GTFs were prepared to evaluate the effect of Quercitrin, DNJ and their combination (Q+D) on its activity.To measure the glucosyltransferase activity, thelevel of water-soluble polysaccharide/glucan (WSP) and alkali soluble polysaccharide/glucan (ASP) formation by crude GTFs was measured.The cell-free enzymes were precipitated from culture supernatant of *S. mutans* by adding solid ammonium sulphate to 70% saturation and were then recovered, as detailed elsewhere [31].This crude enzymatic preparation was stored at -70 °C and was further used for synthesis of water soluble and insoluble glucan. The standard reaction mixture was composed of: 0.25 ml of crude enzyme and sub- MIC levels of Quercitrin, DNJ and Q+D in 20 mM phosphate buffer (pH 6.8),containing 0.25 ml of 0.4 M sucrose. This reaction mixture was incubated at 37 °C for 18 h. Total amount of water soluble and alkali soluble polysaccharide was measured by the phenol–sulphuric acid method [32].Three replicates were made for each concentration of each compound and their combination.

### Effect on sucrose-dependent and sucrose-independent adherence to smooth glass surfaces

The anti- adherence effect of the sub-MIC concentrations of the compounds on *S. mutans* was determined as inhibition of attachment of cells on smooth glass surface. This adherence assay was performed by a method reported earlier with slight modifications [33].The bacteria were grown at an angle of 30 degree for 24 h at 37 °C in a glass tube containing 10 ml of BHI with or without 5% (w/v) sucrose and sub-MIC concentration of Quercitrin, DNJ and Q+D. The solvent controls included BHI with sucrose dependent (SD) and sucrose independent (SI) and equivalent amounts of DMSO and ethanol. The glass tubes, after incubation were gently rotated and the planktonic cells were decanted. The adhered cells were removed by adding 0.5 M of sodium hydroxide followed by vortexing. The cells were washed and suspended in saline. The adherence was estimated spectrophotometrically at 600 nm. All these experiments were performed in triplicates. The untreated BHI medium was taken as control.

Percentage adherence  =  (O.D. of adhered cells/O.D. of total cells) ×100.

### Effect on cell surface hydrophobicity

The estimation of cell surface hydrophobicity of *S. mutans* was done according to Microbial adhesion test to hydrocarbon. Briefly, cells were grown in BHI medium in absence of sucrose with sub- MIC concentration of the compounds (Quercitrin, DNJ and Q+D).These cells were washed twice following by their suspension in sterile saline (0.85%) so that their absorbance was 0.3 at 600 nm. The cell suspension (3.0 ml) was placed in tubes and 0.25 ml of toluene was added. The tubes were agitated in a vortex for 2 min and were allowed to equilibrate for 10 min at room temperature. After toluene phase separation from the aqueous phase, the O.D. of the aqueous phase was determined spectrophotometrically at 600 nm. All determinations were performed as triplicates and controls consisted of cells incubated with or without ethanol. *S. mutans* with a hydrophobic index >70% was arbitrarily classified as hydrophobic [1].

The hydrophobicity was calculated as: (OD initial – OD final)/OD initial ×100%.

### Biofilm formation assay

The biofilm formation was estimated by using the protocol reported earlier with slight modifications [34].It was carried out in flat bottomed 96- wells microtitre plates. Briefly, 50 μL of overnight culture of *S. mutans* was diluted to reach absorbance 0.8 at OD6_00_)and inoculated into 150 ml of BHI containing 5% sucrose with sub- MIC concentration of purified compounds (Quercitrin, DNJ and their combination Q+D) with respective controls (untreated). This suspension (200 μl) was added in the wells and incubated. After incubation at 37 °C for 24 h, the planktonic cells were decanted from the microtitre plates. The remaining unattached cells were removed by gentle flushing with sterile water. The adhered biofilms in the wells were fixed with formalin (37%, diluted 1:10) and 2% sodium acetate. Each well was stained with 200 μL of 0.1% Crystal Violet at room temperature for 15 min. Following at least two rinses with distilled water, bound dye was removed from the cells using 100 μL of 95% ethanol. Plates were then subjected to gentle shaking for 5 min allowing complete release of the dye. Biofilm formation was then quantified by measuring optical density of the suspension at 600 nm by a microplate reader (BIORAD iMark TM Microplate reader, India). These biofilms were formed at 6, 12, 20 and 24 h in the presence of compounds for evaluating time as well as growth phase dependent effects [12].

### Effect on surface rpotein antigen orspaP (or Ag I/II)

The total cellular protein from *S. mutans* was conjugated to rabbit anti-Ag I/II to compare and estimate the levels of Ag I/II protein in treated (with sub- MIC levels of Quercitrin, DNJ and Q+D)and untreated samples. The amount of protein Ag I/II from both the samples was calculated. 10 μg of total protein prepared from untreated and treated*S. mutans* cells wasdissolved in 100 μl of 20 mM carbonate buffer of pH 9.3 was coated on the polystyrene plates. The plates were washed twice with PBS-Tween and were blocked by using 5% skimmed milk in bicarbonate buffer. The plates were washed again with PBS-T thrice and then incubated with rabbit polyclonal Ag I/II antibody at 37 °C for 2 hours. The plates were again washed with PBS thrice and incubated again for 2 hours with 100 μl of anti-rabbit peroxidase coated antibody. The dilutions ranged from 1:100 to 1:1000000 although antibody titer was 1: 10000. It again follows the washing of plates with PBS. The plates were washed thrice with PBS and the 50 μl of TMB (3, 3′, 5, 5′- tetramethylbenzidine). The reaction was immediately stopped after appearance of color using 50 μl of 4 N H_2_SO_4_ [1].

### RNA isolation and quantitative real-time PCR (qRT-PCR)

To study the effect of the sub- MIC levels of Quercitrin, DNJ and Q+D on the expression of virulence genes of *S. mutans*, the organism was cultured in BHI medium supplemented with sub MIC concentration of the compounds to be tested. Bacteria culture (OD_600_ = 0.8) were diluted at a ratio of 1:50 followed by their inoculation into BHI media and were incubated at 37 °C for an overnight growth. RNA was isolated and was purified using Tri-Reagent (Sigma–Aldrich, St. Louis, MO, USA). A reverse transcription (RT) standard reaction mixture (total volume of 20 μL) containing 10 mM dNTPs mix, 20 ng of random hexamers and 1 mg of the total RNA sample was incubated for 5 min at 65 °C to eliminate any secondary structure if present and was finally placed on ice. Then 10X RT buffer, 0.1 M DTT, 25 mM MgCl_2_, 40 U of RNaseOUT Recombinant Ribonuclease Inhibitor and 50 U of Super Script II RT (Invitrogen, Life Technologies, Carlsbad, California, USA) were added to each reaction mixture. After incubation for 10 min at 25°C, the mix was again incubated for 50 min at 42 °C. The mixture was heated for 15 min at 70 °C to terminate the reaction. The cDNA samples were stored at −20 °C until used. The *relA, vicR, brpA, gtfC, covR, spaP, gbpB, comDE* and *smu630* primers were designed using the algorithms provided by Primer Express (Applied Biosystems) for uniformity in size (<95 bp) and melting temperature. Two unrelated genes *GyrA* and *FtsZ* were also used to validate the results. The primer sequences are provided in table 1. PCR conditions included an initial denaturation at 95 °C for 10 min, followed by a 40-cycle amplification consisting of denaturation at 95 °C for 15 s and annealing and extension at 60 °C for 1 min. The expression levels of all the tested genes were normalized using the 16 s rRNA gene of *S. mutans* as an internal standard [1].

**Table 1:** Nucleotide sequences of primers used in this study.

Gene*	Description	Primer sequence (5′ – 3′)	
		Forward	Reverse
*relA*	Guanosine tetra (penta)-phosphatesynthetase	ACAAAAAGGGTATCGTCCGTACAT	AATCACGCTTGGTATTGCTAATTG
*vicR*	Two-component regulatory system	TGACACGATTACAGCCTTTGATG	CGTCTAGTTCTGGTAACATTAAGTCCAATA
*brpA*	Biofilm-regulatory protein	GGAGGAGCTGCATCAGGATTC	AACTCCAGCACATCCAGCAAG
*gtfC*	Water soluble and insoluble glucan production	GGTTTAACGTCAAAATTAGCTGTATTAGC	CTCAACCAACCGCCACTGTT
*covR*	Global response regulator	ACACGATTACAGCCTTTGATGG	CTTCTTAGCCACTTCAAGACC
*spaP*	Cell surface antigen, SpaP (or Ag I/II)	GACTTTGGTAATGGTTATGCATCAA	TTTGTATCAGCCGGATCAAGTG
*gbpB*	Glucan binding protein	ATGGCGGTTATGGACACGTT	TTTGGCCACCTTGAACACCT
*comDE*	Competence-stimulating peptide	ACAATTCCTTGAGTTCCATCCAAG	TGGTCTGCTGCCTGTTGC
*Smu630*	Biofilm-formation hypothetical protein	GTTAGTTCTGGTTTTGACCGCAAT	CCCTCAACAACAACATCAAAGGT
*GyrA*	Unrelated to *S. mutans*	TTCGTACAAGTGTTGCCGATATCT	TCTAGGCGCATCACTTTGACA
*FtsZ*	Unrelated to *S. mutans*	CTGAGATGCCTGCTGCTGAA'	GATTGCTGTGGCTCAGATGATG

* Based on S. mutans genome database (NCBI).

### Target preparation

Crystal structure of C-terminal region of *S. mutans* Antigen I/II was downloaded from protein databank (PDB ID: 3QE5). All water molecules were removed and hydrogen atoms were added by using discovery studio visualizer. Program Q-Site Finder was used for active site detection [35].

### Docking Analyses

Two dimensional structures that were used in the study of binding orientation of selected compounds (Quercitrin, DNJ and their cluster Q+D) into the *S. mutans*surface antigen Ag I/II, were downloaded from Pubchem database. Docking of selected compounds was performed by using GOLD 5.0 [36]. The default parameters of the automatic settings were used to set the genetic algorithm parameters. The protein-ligands complexes were compared based on the scoring function of GOLD fitness score.

### Scanning electron microscopy

The effect of Quercitrin, DNJ and Q+D on biofilm architecture and on the production of extracellular polysaccharide (EPS) or glucan was also determined by scanning electron microscopy (SEM). The cells were grown on sterile glass coverslips by immersing them in 12-well cell culture plate. Sub-MIC concentration of the test compound was taken while the control remained untreated [12]. The wells were inoculated and incubated at 37 °C for 24 h. The coverslips were removed after 24 h and washed three times in sterile PBS. The resultant samples were fixed 2.5% glutaraldehyde in PBS (pH 7.4) with 2% formaldehyde overnight. Post fixing, samples were rinsed thrice with PBS and dehydrated in absolute ethanol series (ethanolic dehydration). The experiment was done in triplicates. Samples were then completely dried, coated with gold, and observed under scanning electron microscope.

### Statistical analysis

All experiments were performed in triplicate and reproduced at least three separate times. Differences between the treated and untreated (control) groups were investigated by SPSS (version 20.0 for Windows). One-way analysis of variance (ANOVA) was performed and a post hoc Tukey's HSD test was used for the comparison of multiple means. Significance was set as a *p* value of<0.05 and *p* value of<0.01.

## Results

### Effect on bacterial viability and growth of *S. mutans*

The minimum inhibitory concentration (MIC) of Quercitrin and Deoxynojirimycin (DNJ) against *S. mutans* was found to be 64 μg/ml and 16 μg/ml respectively. Hence, they were found to have significant antimicrobial activity. Inoculum from individual well without any visible growth was sub-cultured on tryptic soya agar plates. The maximum concentration that completely inhibited the growth (MBC) on the plate was found to be 128 μg/ml and 32 μg/ml for Quercitrin and DNJ respectively.

### Synergistic activity between Quercitrin and Deoxynojirimycin (DNJ) by checkerboard assay

The corresponding fractional inhibitory concentration index (FICI) was found to be 0.313 for Quercitrin and DNJ. These results demonstrate a promising synergistic combination as FICI≤0.5.

### Agreement of synergy by time kill assay

**Figure 1:**
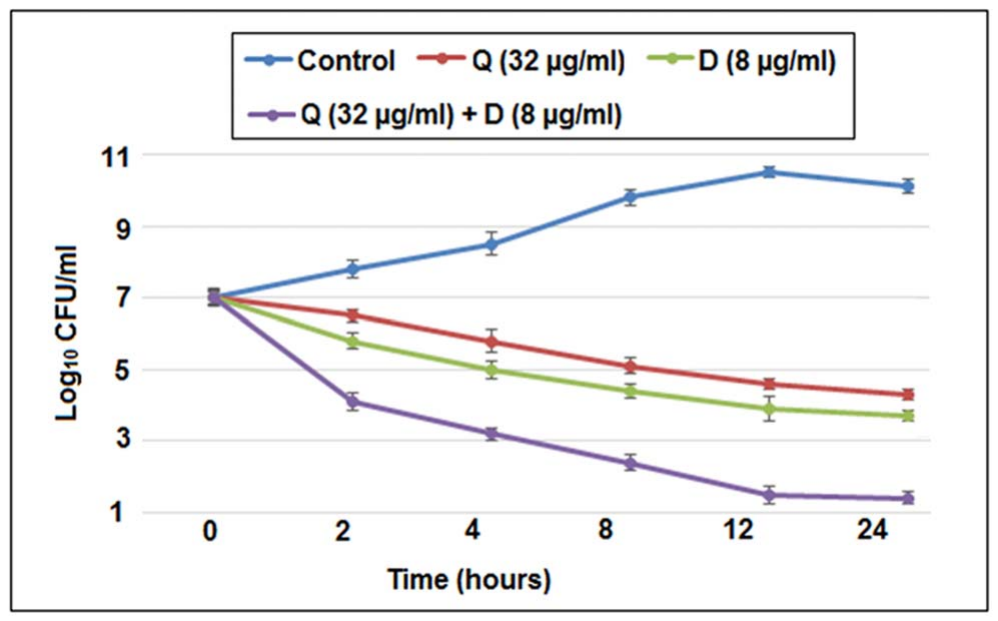
Time kill curves of *S. mutans* incubated with sub- MIC levels of Quercitrin (Q), Deoxynojirimycin (D) and their combination (Q+D). Data represent mean ± SD (n = 3).Significant difference compared with the untreated control (*P*<0.05).

A time-kill experiments were performed to further confirm the synergy between Quercitrin and DNJ (Figure 1). It was observed that with an initial inoculum of 10^7^ CFU/ml, Quercitrinand DNJ alone (sub- MIC level) declined the bacterial growth as the incubation time increases. However, the combination Q+D declined the growth right from the initial hours of incubation and finally yielded more than 2-log_10_ decrease in CFU/ml compared to DNJ (single active agent) after 24 hours of incubation (Figure 1). Such a difference in log_10_ CFU/ml is corresponds to synergistic activity. (Tukey test, p<0.05).

### Synergistic effect of Q+Don acid production and stress tolerance in *S. mutans*

**Figure 2:**
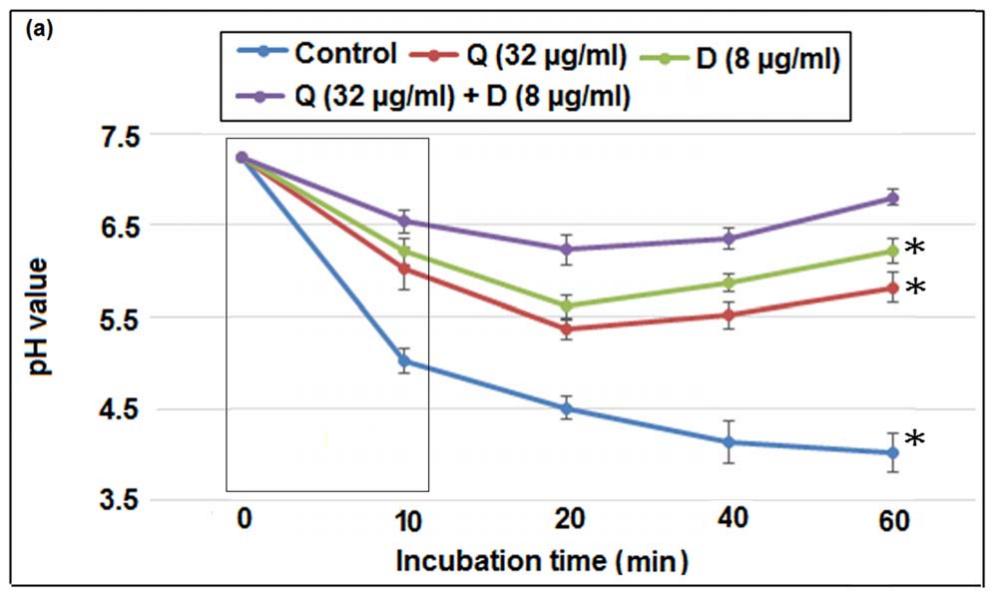
Effect of sub-MIC levels of Quercitrin (Q), Deoxynojirimycin (D) and the combinational effect of these compounds (Q+D) on glycolyticpH-drop (the values enclosed in box corresponds to the initial rate of the pH drop). Data represent mean ± SD (n = 3). *, Significant difference compared with the untreated control (*P*<0.05).

As shown in Figure 2, the glycolytic acid production of *S. mutans*was significantly inhibited by Quercitrin and DNJ individually as well as in combination (Q+D) at sub- MIC levels. In control, the onset pH 7.25 was decreased to 4.01 after 60 min of incubation. Individually, Quercitrin and DNJ efficiently increased this acidic pH (4.01) to 5.82 and 6.22 respectively. However, the most significant change from acidic to near alkaline pH after 1 hour of incubation was observed by the synergistic combination of Q+D, where the acidic pH 4.01 was increased to 6.8. Above all, the initial pH drop from pH 7.25 to 5.02 (maximum) and 6.54 (minimum) was shown by control and combination Q+D respectively. (Tukey test, p<0.05).

The inhibition of F_1_F_o_- ATPase activity of *S. mutans* cells was shown in Figure S1 (a) in file S1. At sub- MIC levels, Quercitrin and DNJ individually inhibited the F_1_F_o_- ATPase activity by25% and 38% respectively. However, their synergistic combination Q+D dramatically reduced the F_1_F_o_- ATPase activity to more than 70% as compared to the vehicle control. (Tukey test, p<0.01). Figure S1 (b) of file S1 shows the inhibition of LDH activity. The protein concentration for control sample was4.871 mg/mland for samples treated with Quercitrin, Deoxynojirimycin and Q+ D were 3.761 mg/ml, 3.512 mg/ml and 3.623 mg/ml respectively.

Quercitrin and DNJ individually reduced LDH activity to only 19% and 21% respectively. However, their combinational effect of Q+D reduced the same to more than 50% as compared to control. A similar pattern in reduction was obtained for enolase activity as shown in Figure S1 (c) in file S1. Quercitrin and DNJ showed a reduction of 18% and 30% respectively. However, their combination acted synergistically as an inhibitor of the glycolytic enzyme enolase by reducing its activity to more than 55%. (Tukey test, p<0.05).

### Inhibition of glucan synthesis, adherence and hydrophobicity

**Figure 3:**
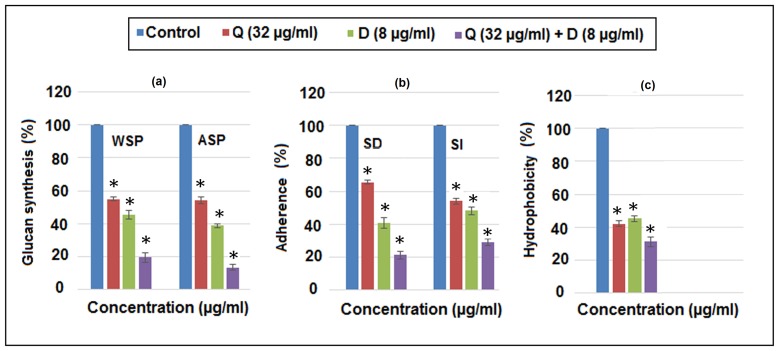
Inhibitory effect of Quercitrin (Q), Deoxynojirimycin (D) and their combinational effect (Q+D) on (a) synthesis of water soluble polysaccharides (WSP) and alkali soluble polysaccharides (ASP) (b) glass-dependent adherence in the absence(sucrose-independent - SI) and presence of 5% sucrose (sucrose-dependent - SD) & (c) cell surface hydrophobicity by *S. mutans*at sub-MIC levels. Data are means ± SD (n = 3).*,Significant difference compared with the untreated control (*P*<0.05).

Figure 3 shows substantial reduction in (a) the synthesis of both water soluble and insoluble glucans by crude GTFs from *S. mutans*, (b) sucrose dependent (SD) and sucrose independent (SI) adherence & (c) hydrophobicity,at sub- MIC level of Quercitrin and DNJ as single agent and in combination (Q+D). Quercitrin inhibited the formation of water soluble polysaccharide (WSP) and alkali soluble polysaccharide (ASP) to approximately 50% whereas DNJ inhibited the synthesis WSP and ASP by >50% and >60% respectively compared to the control. However, the most effective reduction of WSP and ASP was observed by Q+D with a percent reduction of 80% and >85% respectively. Nevertheless, the reduction was consistently observed to be more significant in case of alkali soluble polysaccharide compared to water soluble polysaccharide.

Furthermore, Quercitrin supressed SD and SI adherence to 34.6% and 46.11% respectively. Evidently, SI adherence was more reduced than SD adherence. On the other hand, DNJ inhibited SD and SI adherence by approx. 60% and 52% respectively. Whereas, Q+D displayed an incredible reduction of SD and SI adherence (∼80% and 70% respectively). Hence in contrast to Quercitrin, both DNJ and Q+D reduced SD adherence more than SI adherence.

Figure 3c shows the reduction in hydrophobicity. A percent reduction of 51.22%, 58.48% and 75.88% by Quercitrin, DNJ and Q+D respectively at sub- MIC level was observed. Evidently, the reduction pattern observed was DNJ> Quercitrin > Q+D. Explicitly, DNJ reduced the cell surface hydrophobicity more thanQuercitrin whereas maximum reduction in the hydrophobicity was by their combination Q+D. (Tukey test, p<0.05).

### Reduction of biofilm formation

**Figure 4:**
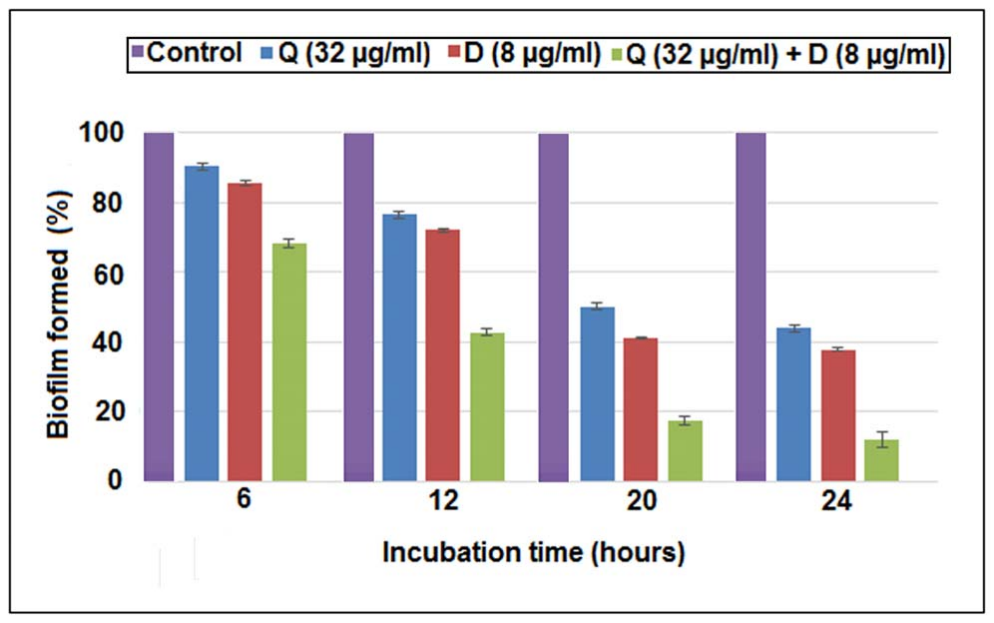
Effect of Quercitrin (Q) and Deoxynojirimycin (D) individually and in combination (Q+D) on the biofilm formation at various growth phases of *S. mutans* at sub-MIC levels. The data represent an average of triplicate experiments ± SD (n = 3).Significant difference compared with the untreated control (*P*<0.05).

The results in Figure 4 demonstrated the anti- biofilm effect of Quercitrin, DNJ and Q+D at different growth phases of biofilm.It attains adherent phase and active accumulated phase at 6 h and 12 h respectively. Whereas, it reaches initial plateau accumulated phase and plateau accumulated phase at 20 h and 24 h respectively. It was consistently seen that the most significant reduction was by the combination of Q+D in all the growth phases. The percentage of adherent cells under control conditions and with different treatments was found to be insignificantly reduced at 6 h of biofilm growth. Whereas a reduction of 25.84% and 37.9% by Q and D respectively was observed at 12 h which was slightly less than the control. However, their combination (Q+D) reduced the biofilm formation to61.5% compared to the control. The adherent cells were quite significantly reduced at 20 and 24 h of *S. mutans* biofilm growth phase. The maximum reduction in biofilm formation was obtained at 24 h in presence of Quercitrin, DNJ and Q+D (57.3%, 70% and 85% respectively) as compared to the control. Hence, biofilm formation was inhibited during the active accumulated phase, initial plateau accumulated phase and plateau accumulated phase. (Tukey test, p<0.05).The absolute value of biofilm obtained (in terms of absorbance) for the control group were 0.368, 0.673, 0.818 and 1.414 for 6 h, 12 h, 20 h and 24 h respectively.

### Reduction of surface protein antigen (SpaP/Ag I/II)

**Figure 5:**
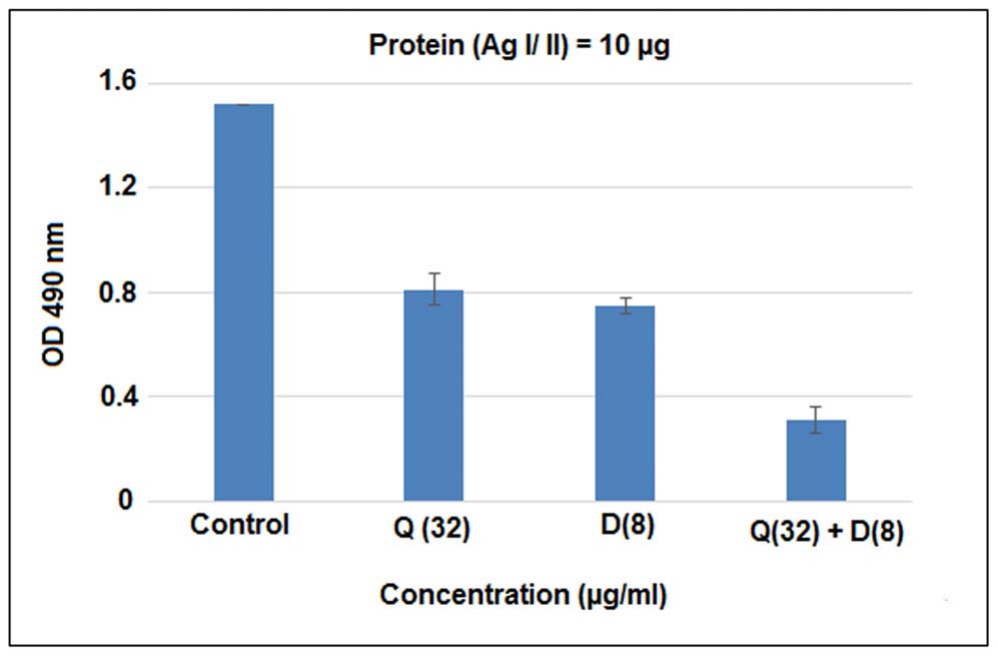
Direct binding ELISA of total protein from untreated sample and samples treated with Quercitrin (Q) and Deoxynojirimycin (D) individually and their combination (Q+D) against polyclonal antibodies of Ag I/II. Data are means ± SD (n = 3).

The reduction of surface protein antigen Ag I/II or spa P in samples treated with Quercitrin, DNJ and the combination (Q+D) was found to be 40% (OD 1.52), 50% (OD 0.75) and 78.2% (OD 0.31) respectively at 1:100 antibody dilution as compared to the control (Figure 5).The protein concentrations obtained were; control (5.015 mg/ml) and for treated samples; Quercitrin (4.530 mg/ml), Deoxynojirimycin (4.316 mg/ml) and Q+ D (4.395 mg/ml).

### Expression profile of virulence genes

**Figure 6:**
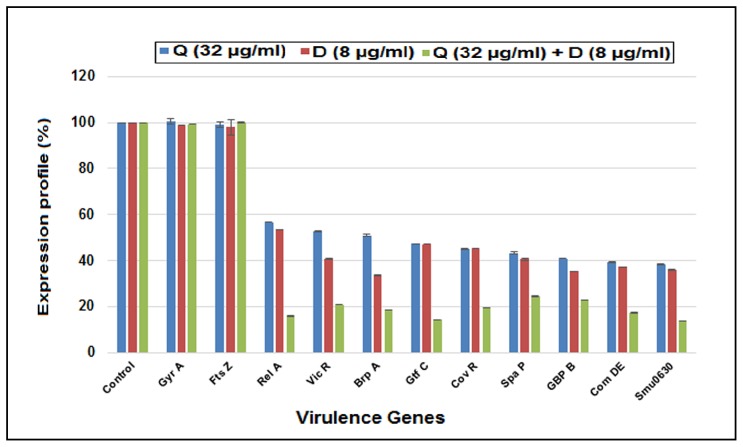
Expression profile of various virulence genes of *S. mutans* in response to the treatment with sub- MIC levels of Quercitrin (Q), Deoxynojirimycin (D) and their combination (Q + D). Data presented were generated from at least three independent sets of experiments (Data  =  mean ± SD).Significant difference compared with the untreated control (*P*<0.05).

The expression profile of different virulence genes (*relA, vicR, brpA, gtfC, covR, spaP, gbpB, comDE* and *smu630*) of *S. mutans* treated with Quercitrin, DNJ and their combination Q+D was determined (Figure 6). The entire set of virulence genes was found to be suppressed after treatment with each compound. Whilst the combination Q+D showed incredible reduction in the expression of all genes. The expression of *relA, brpA, gtfC, covR, comDE* and *smu630* genes was maximally reduced by >80% whereas, the expression *of vicR, spaP* and *gbpB* was suppressed by >75% as compared to the control. Moreover, Quercitrin suppressed the expression level of *relA, vicR, brpA, gtfC, covR, spaP, gbpB, comDE* and *smu630* by 43.34%, 47.4%, 49.07%, 53%, 54.1%, 56.74%, 59.07%, 60.87% and 61.79% respectively. Likewise, DNJ repressed the expression level of *relA, vicR, brpA, gtfC, covR, spaP, gbpB, comDE* and *smu630* by 46.6%, 59.31%, 66.44%, 53%, 54.87%, 59.49%, 64.77%, 62.82% and 64.2% respectively as compared to the control. Evidently, both Quercitrin and DNJ suppressed *gtfC* and *covR* gene by 53% and 54% respectively. However, the expression data of the two unrelated genes *GyrA* and *FtsZ* showed no significant effect of the compounds used individually (Quercitrin and DNJ) or in combination (Q+D) as shown in Figure 6 (Dissociation curves of unrelated genes are given in Figure S2 in file S1). Hence, validating their specific activity against genes involved in *S. mutans* pathogenesis.

### Docking analyses

**Figure 7:**
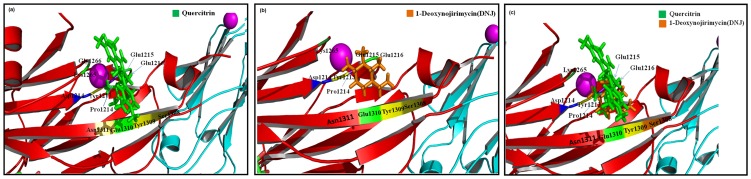
Binding pattern of (a) Quercitrin (Q) (b) Deoxynojirimycin (D) (c) Quercitrin & Deoxynojirimycin as cluster (Q + D) within the active site of Ag I/II.

Quercitrin and DNJ were found to interact with a Gold fitness score of 65 and 68 respectively. Eleven amino acids (Asn1311, Glu1215, Glu1216, Glu1310, Tyr1213, Tyr1309, Ser1308, Pro1214, Asp1211, Lys1265 and Gly1266) were common for both the ligands binding and stabilizing the complex as shown in Figure 7.

### Inhibition of EPS synthesis and impairment of biofilm architecture visualized by scanning electron microscopy

**Figure 8:**
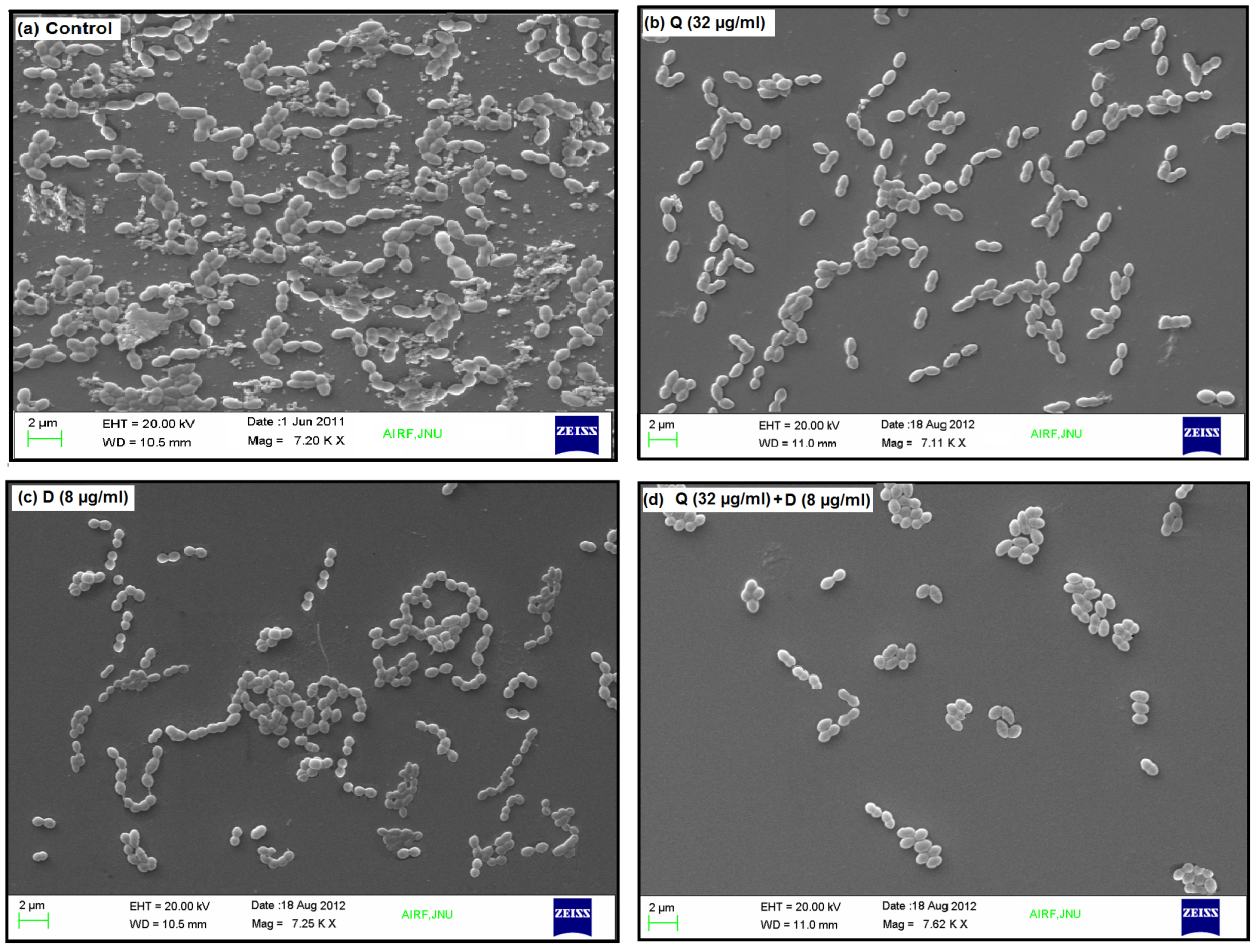
Scanning electron micrograph of *S. mutans* biofilm formed after24 h of incubation. **(a)** Control and in the presence of sub- MIC levels of **(b)** Quercitrin (Q), **(c)** Deoxynojirimycin (D) &**(d)** and their combination (Q + D).

Scanning electron microscopy depicted the impact of the Quercitrin, DNJ and Q+D on the activity of *S. mutans* to synthesize extracellular polysaccharides (Figure 8 b, c & d). In concordance with the aforesaid results about reduction in glucan synthesis, adherence, biofilm formation and *gtf* suppression, the treated group displayed significant dispersion of the cells suggesting reduced amount of exopolysaccharide synthesis. Conversely, the control sample (Figure 8a) showed clear aggregates of cells immersed into the exopolysaccharide pool.

## Discussion

The role of *S. mutans* in the pathogenesis of caries is well documented. As mentioned, it establishes the infection by numerous virulence characteristics like biofilm formation, acidogenicity, aciduracity, adherence, glucan synthesis, cell surface hydrophobicity and quorum sensing mechanism. Hence, suppressing the genes and enzymes associated with these virulence mechanisms could be an appealing approach for the prevention of *S. mutans* persistence and pathogenesis. The current study, to the best of our knowledge, by far, is the first report providing strong evidence that Quercitrin in combination with Deoxynojirimycin (DNJ) is synergistic across the range of cariogenic mechanisms of *S. mutans* compared to their individual effect. The trend however, is consistent throughout the study, i.e., the synergistic combination shows the best anti- cariogenic activity followed by DNJ and finally Quercitrin.

Quercitrin and DNJ were found to have a significant antimicrobial activity with low MIC values of 64 μg/ml and 16 μg/ml respectively. The MIC value of DNJ is in consistent with the earlier data from our group [12].The results obtained from checkerboard and time kill assay were in concordance with each other suggesting promising synergistic activity against *S. mutans*.

Acidogenesis (acid production) and aciduracity (acid tolerance) are key cariogenic virulence factors of *S. mutans* [37]. Bearing these properties, *S. mutans* has an upper hand over less-acid-tolerant species and hence impose physiological stress on them. Thus, even in stress conditions, it emerges out to be most prevalent inhabitant of cariogenic plaque. Thus, stress tolerance plays a crucial role in its pathogenesis.

A study has shown that, the cariostatic effect can be attained by reducing the bacterial acidogenesis or by inhibiting the activity of enzyme associated with the glycolysing systems of *S. mutans* [1]. Hence, following are the results obtained against various acid producing mechanisms and the inhibition of glycolytic enzyme.

The results obtained from glycolytic pH drop showed tremendous reduction in the initial and final rate of the pH drop suggesting the impairment of acid production capability of *S. mutans* cells. This combinational effect may be due to the suppression of bacterial glycolytic pathway. Since, resting bacteria were used for this assay, chance of inhibition by the reduction of *S. mutans* growth is ruled out. Furthermore, since the final pH values in glycolytic pH-drop assay implies acid tolerance [38], the apparent reduction in their values signifies the disruption of aciduracity as well.

Although, the acid tolerance of *S. mutans* is mainly credited to the activity of F_1_F_o_- ATPase as it maintains pH gradient across the membrane [39]. It is evident from the data obtained, that the synergistic combination of Quercitrin and DNJ showed remarkable reduction in the activity of F_1_F_o_- ATPase system as well. Thus, the forbiddance of F_1_F_o_-ATPase enzymatic activity may contribute to rise in cytoplasmic acidity resulting in decreased acid adaptation [9]. Since, cytoplasmic alkalization is important for normal functioning of a series of enzymes involved in physiological processes like glycolysis, cell persistence, IPS & EPS production, its acidification can lead to potential mortal effect on *S. mutans*.

Moreover, the synergistic combination Q+D has also suppressed the lactate dehydrogenase (LDH) activity at enzymatic level, *in vitro*. LDH is known for lactic acid production and is also involved in *S. mutans* pathogenicity [40].The inhibition of LDH at enzymatic levels may result in increased levels of NADH, leading to a drop in the redox potential of the cell, resulting in NAD^+^/NADH imbalance and accumulation of glycolytic intermediates in the cell, which is identified to be toxic for *S. mutans* [9], [10]. Hence, it would result in impaired cytoplasmic alkalization and interrupted glycolysis with reduced ATP pools, leading to compromised adaptation to environmental stress and impaired cellular functions, resulting even in cell death. Moreover, we have found that the combination Q+D significantly inhibited the activity of glycolytic enzyme enolase, in turn inhibiting the bacterial glycolytic pathways as shown. Enolase is responsible for the formation of phosphoenolpyruvate (PEP), which functions as the vital component of the PEP: carbohydrate phosphotransferase system (PTS) [9], which is a key system for internalizing sugar into the cell throughout depleted sugar state [41]. Hence, the inhibition of enolase activity will not only result in reduced glycolysis but will also reduce the downstream production of phosphoenolpyruvate (PEP) leading to decreased acidogenesis. Hence, this study confirms that the *in vitro* inhibition of acid production by *S. mutans* is as a result of suppression of multiple biomolecular mechanisms involved in the bacterial acidogenesis.

Furthermore, GTF plays a crucial role in the conversion of sucrose to water soluble and insoluble polysaccharides (glucans). These glucans mediate the adherence of *S. mutans* on the tooth surface. However, it is well demonstrated that water-insoluble glucans(also known as alkali soluble polysaccharides) synthesized by GTFs play more critical role in facilitating the attachment and colonization of *S. mutans* in the oral cavity [42]. It is evident from the data that there was an exemplary reduction in the synthesis of both, water soluble as well as alkali soluble polysaccharide. However, the reduction of alkali soluble polysaccharide was more pronounced and will result in significant suppression of adhesive interactions leading to unsuccessful adherence and impaired biofilm formation.

Adherence, marks another decisive factor in cariogenesis by *S. mutans* and therefore, preventing the adherence of bacteria could be a strong step of prevention of its pathogenesis [1].Sucrose-independent (SI) adherence is is associated with hydrophobic interactions between the cells and the tooth surface. The reduced hydrophobic interactions will inevitably lead to reduction in SI adherence. Sucrose dependent (SD) adherence, on the other hand, is mediated by bacterial synthesis of glucans from sucrose which helps in the formation of sticky clumps by the cells [12].

The cell surface hydrophobicity of *S. mutans* also showed apparent reduction. Besides, the cell-surface hydrophobicity of *S. mutans* is known to be associated with cell-surface proteins [43]. The potential rationale of this reduction in hydrophobicity could be hypothesised as a result of binding of these active compounds (Quercitrin and DNJ) to different proteins associated with the cell surface (Ag I/II).

It has also been proved from the data that Quercitrin reduced hydrophobicity to a greater extent than DNJ and therefore, the reduction in SI adherence was more pronounced by Quercitrin than DNJ. Whereas, DNJ reduced the glucan formation more than Quercitrin and hence showed a greater reduction of SD adherence as compared to SI adherence. However, Q+D, showed a cumulative activity by reducing all these parameters to a much pronounced level than they reduced individually, thereby leading to a remarkable cariostatic effect.

Also, biofilm formation is one of the many mechanisms adopted by *S. mutans* which results in its virulence and is also mediated by glucan. The anticariogenic effect of the synergistic combination of Quercitrin and DNJ was seen as a reduction in the biofilm formation during different growth phases, in order to reflect the inhibitory role in biofilm development and maturation rather than initial adherence to the surface [44]. It was found that there was no significant reduction after 6 h of incubation which marks the initial attachment (or primary adherent phase). However, significant reduction was obtained at 12 h (active accumulated phase) and thereafter on 20 h (initial plateau accumulated phase) and 24 h (plateau accumulated phase) of incubation, implying inhibition of biofilm maturation at crucial developmental stages.

Antigen I/II (also known as Ag I/II, P1 or SpaP) is a surface anchored protein utilized by *S. mutans* for adhesion to the tooth surface. Apart from adhesion, AgI/II is involved in biofilm formation, promotes platelet aggregation, collagen-dependent bacterial invasion of dentin and finally resulting in cariogenecity [45].In this study, ELISA of total protein was used to compare the level of expression of Ag I/II. It was found that its expression was drastically reduced by the Quercitrin and DNJ in combination as compared to their ondividual effect and to the vehicle control. As, Ag I/II is involved in a number of factors resulting in pathogenesis, its suppression will lead to a cariostatic effect.

Docking studies predict the conformation and the binding affinity of a ligand (Quercitrin/Deoxynojirimycin) against a target protein (Ag I/II or SpaP). Quercitrin and DNJ were able to make stable conformations within the active sites of Ag I/II with a Gold fitness score of 65 and 68 respectively. This, in turn supports the study where DNJ is observed as better anticariogenic agent than Quercitrin. Also, the binding of these compounds in the active site will make Ag I/II unavailable to adhere to the tooth pellicle resulting in impaired adhesion.

Scanning electron microscopy in case of control depicted that the cells were embedded in the sticky exopolysaccharide pool which is known to stimulate cell clumping [1]. However, in treated samples, the integrity of a virulent biofilm was obliterated. There was hardly any trace of exopolysaccharide suggesting the reduction in its synthesis. Also, the cells were clearly scattered without any clumps or aggregate formation.

Finally, the expression profile of selected virulence genes showed that entire set of genes were suppressed in the presence of Quercitrin and Deoxynojirimycin individually. Nevertheless, their synergistic combination showed a tremendous downregulation of genes as compared individual compounds.

Genes like *relA* gene encodes guanosine tetra (penta)-phosphate synthetase and is known to be involved in the acid and oxidative stress tolerance mechanisms of *S. mutans* [46] and *brpA* (biofilm regulatory protein)plays a crucial role in biofilm formation and itsstructural integrity. The suppression of these genes will lead to impaired acid tolerance and major structural defects in biofilm formation andintegrity respectively, resulting in despaired virulence expressions.

Similarly, *smu630*, which is a hypothetical proteinalsocontributes to biofilm formation. Thus, its downregulation will also be an alternative approach to reduce *S. mutans* virulence [46].*VicR* and *CovR* genes in *S. mutans* are known to regulate a panel of genes encoding for synthesis of glucans (*gtfC, gtfD, gbpB*), which are critical for adherence to a smooth tooth surface and essential for virulence [47].The *gbpB* gene (glucan binding protein), has been recognized to have affinity for glucans, thus, promoting bacterial adhesion [1]. In addition, *gtfC*, on the other hand (glucosyltransferases), catalyse the synthesis of water insoluble and alkali soluble glucan from sucrose, which are apparently required for plaque formation and structurally stable biofilms. The reduction in the expression of the aforesaid genes will thereby suppress a series of cascades involved in biofilm formation, cell wall integrity, adhesion promotion and surface biogenesis.

Another gene designated as *spaP* (Ag I/II or P1), a surface protein of the antigen, is critical in *S. mutans* for initial adhesion to the tooth surface [1]. This protein is also involved in biofilm formation, promotes platelet aggregation and collagen-dependent bacterial invasion of dentin [45]. Hence, the suppression of this gene will lead to inhibition of adhesion and may lead to anticariogenic action. Hence, it has observed that both *gtfC* and *spaP* genes are inhibited at transcriptional as well as enzymatic levels.

In addition, *comDE* gene was also repressed dramatically. The regulatory gene *comDE*, is a part of the quorum-sensing cascade of *S. mutans* [46]. The downregulation of this gene will attenuates the internal communication system utilized by the bacteria to alter their gene expression at a critical density of the cell population which may even lead to cell death.

**Figure 9:**
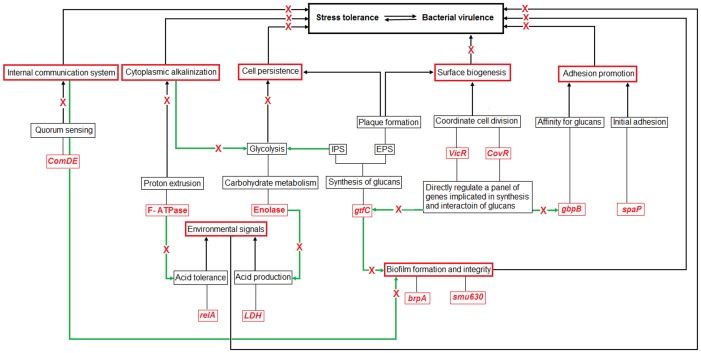
Inhibitory effects of synergistic combination of Quercitrin and Deoxynojirimycin (Q+ D) on different genes and bacterial enzymes involved in regulatory network (stress tolerance mechanisms) resulting in virulence by *S. mutans*. Green arrows represent the interaction among individual virulence mechanisms while red cross represents the inhibition of the physiological processes.

Precisely, all these genes and enzymes are linked to each other at different step of the cascade in regulatory network of *S. mutans*. As summarized in figure 9, the prodigious suppression in the gene expression and enzymatic activity will therefore lead to a state of compromised stress tolerance resulting in a complete shutdown of overall intracellular metabolism of the bacterial cells. Consequently, prohibiting the bacterial pathogenesis.

This study concludes that Quercitrin and DNJ at a described ratio will be an effective combination to suppress the cariogenic pathways of *S. mutans*. Hence, this combination might be proposed as a novel therapeutic agent to inhibit dental caries.

## Supporting Information

File S1 **Contains: Figure S1.** Effect of sub-MIC levels of Quercitrin (Q), Deoxynojirimycin (D) and the combinational effect of these compounds (Q+D) on enzymes associated with the acidurity and acidogenicity of*S. mutans*. **(a)** F- ATPase activity **(b)** LDH activity and **(c)** Enolase activity. The significant difference compared with the control (*P*<0.05). Data represent mean± SD (n = 3). **Figure S2.** The dissociation curves of endogenous control*GyrA* and *FtsZ*are shown in panel **(a)**, **(b)** and **(c)** respectively.
(DOC)

## References

[1] Hasan S, Danishuddin M, Adil M, Singh K, Verma PK, et al (2012) Efficacy of *E. officinalis* on the Cariogenic Properties of Streptococcus mutans: A Novel and Alternative Approach to Suppress Quorum-Sensing Mechanissm. PLoS One 7: e40319. 22792279 10.1371/journal.pone.0040319 PMC3390397

[2] Lévesque CM, Voronejskaia E, Huang YC, Mair RW, Ellen RP, et al (2005) Involvement of sortase anchoring of cell wall proteins in biofilm formation by Streptococcus mutans. Infect Immun 73: 3773–7. 15908410 10.1128/IAI.73.6.3773-3777.2005 PMC1111851

[3] Miller-Torbert TA, Sharma S, Holt RG (2008) Inactivation of a gene for a fibronectin-binding protein of the oral bacterium *Streptococcus mutans* partially impairs its adherence to fibronectin. Microb Pathog 45: 53–9. 18479886 10.1016/j.micpath.2008.02.001 PMC2493617

[4] Nakano K, Nomura R, Nemoto H, Mukai T, Yoshioka H, et al (2007) Detection of novel serotype k *Streptococcus mutans* in infective endocarditis patients. J Med Microbiol56: 1413–5. 10.1099/jmm.0.47335-0 17893184

[5] Quivey RG Jr, Kuhnert WL, Hahn K (2000) Adaptation of oral streptococci to low pH. Adv Microb Physiol 42: 239–74. 10907552 10.1016/s0065-2911(00)42004-7

[6] Schilling KM, Bowen WH (1992) Glucans synthesized in situ in experimental salivary pellicle function as specific binding sites for *Streptococcus mutans* . Infect Immun 60: 284–95. 1530843 10.1128/iai.60.1.284-295.1992 PMC257534

[7] Jeon JG, Rosalen PL, Falsetta ML, Koo H (2011) Natural products in caries research: Current (limited) knowledge, challenges and future perspective. Caries Res 45: 243–263. 21576957 10.1159/000327250 PMC3104868

[8] Kobayashi H, Suzuki T, Unemoto T (1986) Streptococcal cytoplasmic pH is regulated by changes in amount and activity of a proton-translocating ATPase. J Biol Chem 261: 627–30. 2416756

[9] Xu X, Zhou XD, Wu CD (2011) The tea catechin epigallocatechin gallate suppresses cariogenic virulence factors of *Streptococcus mutans* . Antimicrob Agents Chemother 55: 1229–36. 21149622 10.1128/AAC.01016-10 PMC3067078

[10] Chen A, Hillman JD, Duncan M (1994) L-(+)-lactate dehydrogenase deficiency is lethal in *Streptococcus mutans* . J Bacteriol 176: 1542–1545. 8113201 10.1128/jb.176.5.1542-1545.1994 PMC205228

[11] Yamashita Y, Bowen WH, Burne RA, Kuramitsu HK (1993) Role of the *Streptococcus mutans* gtf genes in caries induction in the specific-pathogen-free rat model. Infect Immun 61: 3811–7. 8359902 10.1128/iai.61.9.3811-3817.1993 PMC281081

[12] Islam B, Khan SN, Haque I, Alam M, Mushfiq M (2008) Novel antiadherence activity of Mulberry Leaves: Inhibition of *Streptococcus mutans* Bio-film by 1-Deoxynojirimycin isolated from *Morus alba* . J Antimicrob Chemother 62: 751–757. 18565974 10.1093/jac/dkn253

[13] Service RF (1995) Antibiotics that resist resistance. Science 270: 724–7. 7481757 10.1126/science.270.5237.724

[14] Hasan S, Ali SZ, Khan AU (2013) Novel combinations of antibiotics to inhibit extended-spectrum β-lactamase and metallo-β-lactamase producers *in vitro*: a synergistic approach. Future Microbiol 8: 939–44. 23841638 10.2217/fmb.13.54

[15] Müller P, Alber DG, Turnbull L, Schlothauer RC, Carter DA, et al (2013) Synergism between Medihoney and rifampicin against methicillin-resistant *Staphylococcus aureus* (MRSA). PLoS One 8: e57679. 23469049 10.1371/journal.pone.0057679 PMC3585195

[16] Celenza G, Segatore B, Setacci D, Bellio P, Brisdelli F, et al (2012) *In vitro* antimicrobial activity of pannarin alone and in combination with antibiotics against methicillin-resistant *Staphylococcus aureus* clinical isolates. Phytomedicine 19: 596–602. 22459282 10.1016/j.phymed.2012.02.010

[17] Muzitano MF, Cruz EA, de Almeida AP, Da Silva SA, Kaiser CR, et al (2006) Quercitrin: an antileishmanial flavonoid glycoside from *Kalanchoe pinnata* . Planta Med 72: 81–3. 16450304 10.1055/s-2005-873183

[18] Comalada M, Camuesco D, Sierra S, Ballester I, Xaus J, et al (2005) *In vivo* quercitrin anti-inflammatory effect involves release of quercetin, which inhibits inflammation through down-regulation of the NF-kappaB pathway. Eur J Immuno35: 584–92. 10.1002/eji.200425778 15668926

[19] Muzitano MF, Cruz EA, de Almeida AP, Da Silva SA, Kaiser CR, et al (2006) Quercitrin: an antileishmanial flavonoid glycoside from *Kalanchoe pinnata* . Planta Med 72: 81–3. 16450304 10.1055/s-2005-873183

[20] Hong CO, Lee HA, Rhee CH, Choung SY, Lee KW (2013) Separation of the antioxidant compound quercitrin from Lindera obtusiloba Blume and its antimelanogenic effect on B16F10 melanomacells. Biosci Biotechnol Biochem 77: 58–64. 23291772 10.1271/bbb.120562

[21] Wagner C, Fachinetto R, Dalla Corte CL, Brito VB, Severo D, et al (2006) Quercitrin, a glycoside form of quercetin, prevents lipid peroxidation *in vitro* . Brain Res 30: 192–8. 10.1016/j.brainres.2006.05.084 16828712

[22] Yin Y, Li W, Son YO, Sun L, Lu J, et al (2013) Quercitrin protects skin from UVB-induced oxidative damage. Toxicol Appl Pharmacol 269: 89–99. 23545178 10.1016/j.taap.2013.03.015 PMC3700335

[23] Tsuduki T, Kikuchi I, Kimura T, Nakagawa K, Miyazawa T (2013) Intake of mulberry 1-deoxynojirimycin prevents diet-induced obesity through increases in adiponectin in mice. Food Chem 139: 16–23. 23561072 10.1016/j.foodchem.2013.02.025

[24] Timokhova AV, Bakinovskiľ LV, Zinin AI, Popenko VI, Ivanov AV, et al (2012) Affect of deoxynojirimycin derivatives on hepatitis C virus morphogenesis. Mol Biol (Mosk) 46: 644–53. 23113354

[25] Li YG, Ji DF, Zhong S, Lv ZQ, Lin TB (2013) Cooperative anti-diabetic effects of deoxynojirimycin-polysaccharide by inhibiting glucose absorption and modulating glucose metabolism in streptozotocin-induced diabetic mice. PLoS One 8: e65892. 23755289 10.1371/journal.pone.0065892 PMC3675047

[26] Ban SH, Kim JE, Pandit S, Jeon JG (2012) Influences of Dryopteris crassirhizoma extract on the viability, growth and virulence properties of *Streptococcus mutans* . Molecules 17: 9231–44. 22858843 10.3390/molecules17089231 PMC6268259

[27] Belli WA, Buckley DH, Marquis RE (1995) Weak acid effects and fluoride inhibition of glycolysis by *Streptococcus mutans* GS-5. Can J Microbiol41: 785–91. 10.1139/m95-108 7585355

[28] Bencini DA, Shanley MS, Wild JR, O'Donovan GA (1983) New assay for enzymatic phosphate release: Application to aspartate transcarbamylase and other enzymes. Ana Biochem132: 259–264. 10.1016/0003-2697(83)90005-2 6353998

[29] Bradford MM (1976) A rapid and sensitive method for the quantitation of microgram quantities of protein utilizing the principle of protein-dye binding. Anal Biochem 72: 248–54. 942051 10.1016/0003-2697(76)90527-3

[30] Crow VL, Pritchard GG (1977) Fructose 1, 6-diphosphate-activated L-lactate dehydrogenase from *Streptococcus lactis*: kinetic properties and factors affecting activation. J. Bacteriol. 131: 82–91. 10.1128/jb.131.1.82-91.1977 PMC235394 17595

[31] Koo H, Gomes BP, Rosalen PL, Ambrosano GM, Park YK, Cury JA (2000) In vitro antimicrobial activity of propolis and Arnica montana against oral pathogens. Arch Oral Biol 45: 141–148. 10716618 10.1016/s0003-9969(99)00117-x

[32] Dubois M, Gilles KA, Hamilton JK, Rebers PA, Smith F (1956) Colorimetric method for determination of sugars and related substance. Anal Chem 28: 350–356. 10.1038/168167a0 14875032

[33] Hamada S, Torii M (1978) Effect of sucrose in culture media on the location of glucosyltransferase of *Streptococcus mutans* and cell adherence to glass surfaces. Infect Immun 20: 592–599. 669814 10.1128/iai.20.3.592-599.1978 PMC421899

[34] Loo CY, Corliss DA, Ganeshkumar N (2000) *Streptococcus gordonii* biofim formation: identification of genes that code for biofilm phenotypes. J Bacteriol182: 1374–82. 10.1128/jb.182.5.1374-1382.2000 PMC94426 10671461

[35] Laurie AT, Jackson RM (2005) Q-SiteFinder: an energy-based method for the prediction of protein-ligand binding sites. Bioinformatics 21: 1908–16. 15701681 10.1093/bioinformatics/bti315

[36] Jones G, Willett P, Glen RC (1995) Molecular recognition of receptor sites using a genetic algorithm with a description of desolvation. J Mol Biol 245: 43–53. 7823319 10.1016/s0022-2836(95)80037-9

[37] Kuramitsu HK (1993) Virulence factors of mutans streptococci: role of molecular genetics. Crit Rev Oral Biol Med 4: 159–76. 8435464 10.1177/10454411930040020201

[38] Gregoire S, Singh AP, Vorsa N, Koo H (2007) Influence of cranberry phenolics on glucan synthesis by glucosyltransferases and *Streptococcus mutans* acidogenicity. J Appl Microbiol 103: 1960–1968. 17953606 10.1111/j.1365-2672.2007.03441.x

[39] Hamilton IR, Buckley ND (1991) Adaptation by *Streptococcus mutans* to acid tolerance. Oral Microbiol Immunol 6: 65–71. 1658715 10.1111/j.1399-302x.1991.tb00453.x

[40] Lemos JA, Abranches J, Burne RA (2005) Responses of cariogenic streptococci to environmental stresses. Curr Issues Mol Biol 7: 95–107. 15580782

[41] Postma PW, Lengeler JW, Jacobson GR (1993) Phosphoenolpyruvate:carbohydrate phosphotransferase systems of bacteria. Microbiol Rev 57: 543–94. 8246840 10.1128/mr.57.3.543-594.1993 PMC372926

[42] Yamashita Y, Bowen WH, Burne RA, Kuramitsu HK (1993) Role of the *Streptococcus mutans* gtf genes in caries induction in the specific pathogen free rat model. Infect Immun 61: 3811–7. 8359902 10.1128/iai.61.9.3811-3817.1993 PMC281081

[43] McBride BC, Song M, Krasse B, Olsson J (1994) Biochemical and immunological differences between hydrophobic and hydrophilic strain of Streptococcus mutans. Infect Immun 44: 68–75. 10.1128/iai.44.1.68-75.1984 PMC263470 6706407

[44] Wen ZT, Burne RA (2002) Functional genomics approach to identifying genes required for biofilm development by *Streptococcus mutans* . Appl Environ Microbiol 68: 1196–203. 11872468 10.1128/AEM.68.3.1196-1203.2002 PMC123778

[45] Larson MR, Rajashankar KR, Crowley PJ, Kelly C, Mitchell TJ (2011) Crystal structure of the C-terminal region of *Streptococcus mutans* antigen I/II and characterization of salivary agglutinin adherence domains. J Biol Chem 286: 21657–66. 21505225 10.1074/jbc.M111.231100 PMC3122222

[46] Liu C, Worthington RJ, Melander C, Wu H (2011) A new small molecule specifically inhibits the cariogenic bacterium *Streptococcus mutans* in multispecies biofilms. Antimicrob Agents Chemother 55: 2679–87. 21402858 10.1128/AAC.01496-10 PMC3101470

[47] Stipp RN, Boisvert H, Smith DJ, Höfling JF, Duncan MJ (2013) CovR and VicRK Regulate Cell Surface Biogenesis Genes Required for Biofilm Formation in *Streptococcus mutans* . PLoS One 8: e58271. 23554881 10.1371/journal.pone.0058271 PMC3595261

