# Supplementary figures and images for: Inhibition of Major Virulence Pathways of Streptococcus mutans by Quercitrin and Deoxynojirimycin: A Synergistic Approach of Infection Control
Source: PLoS One. 2014 Mar 12;9(3):e91736. doi: 10.1371/journal.pone.0091736 (PMC3951425; doi:10.1371/journal.pone.0091736)

Figure S1


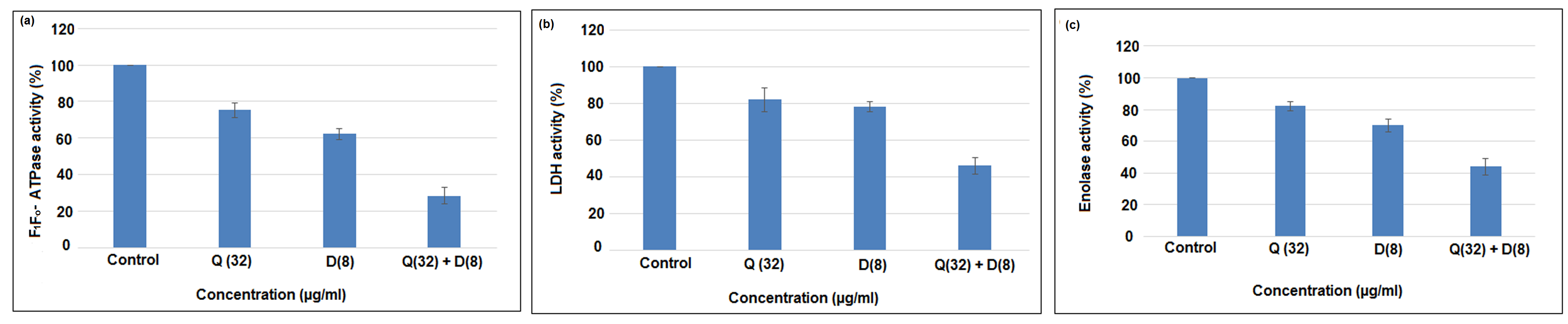


Figure S2 a


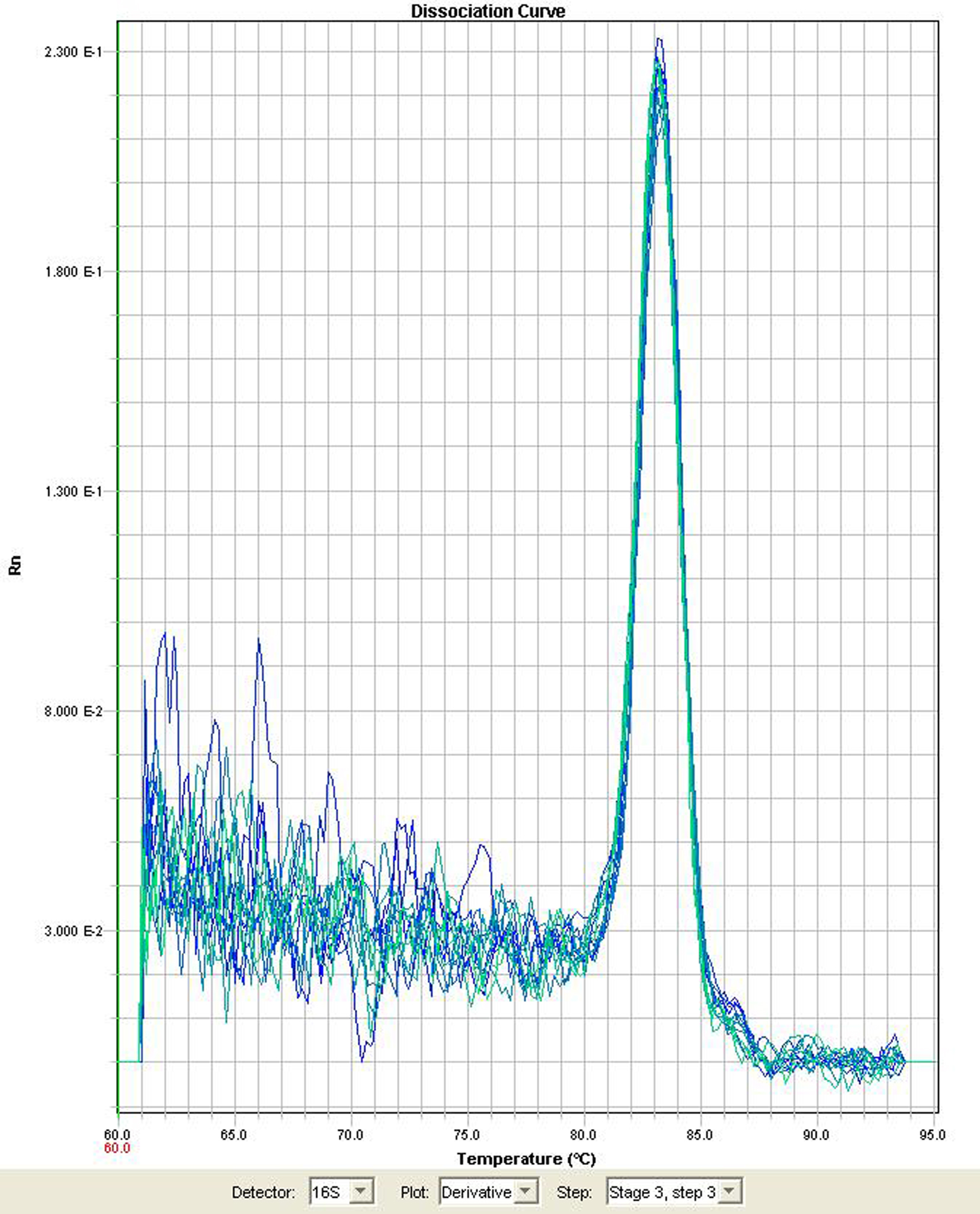


Figure S2 b


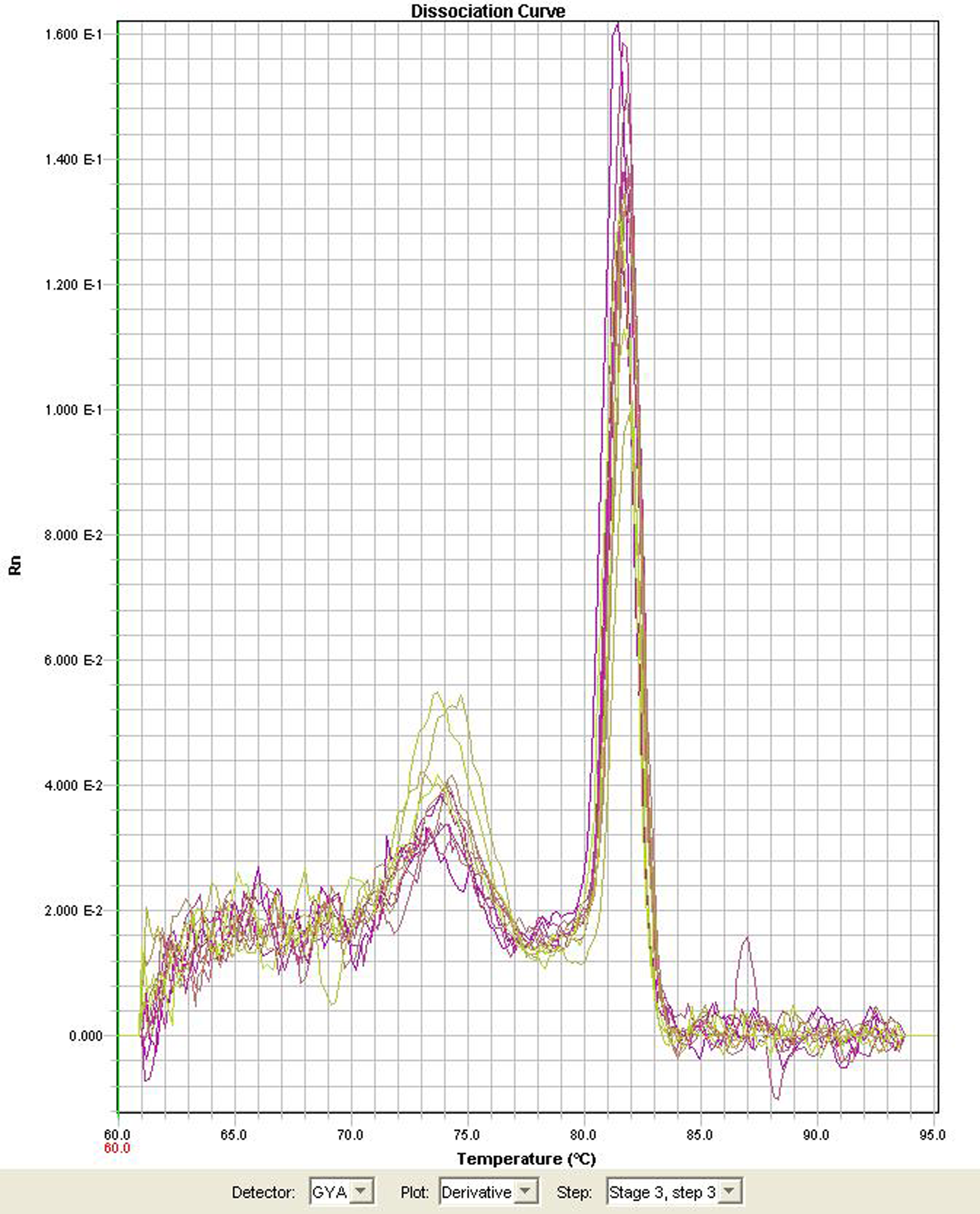


Figure S2 c


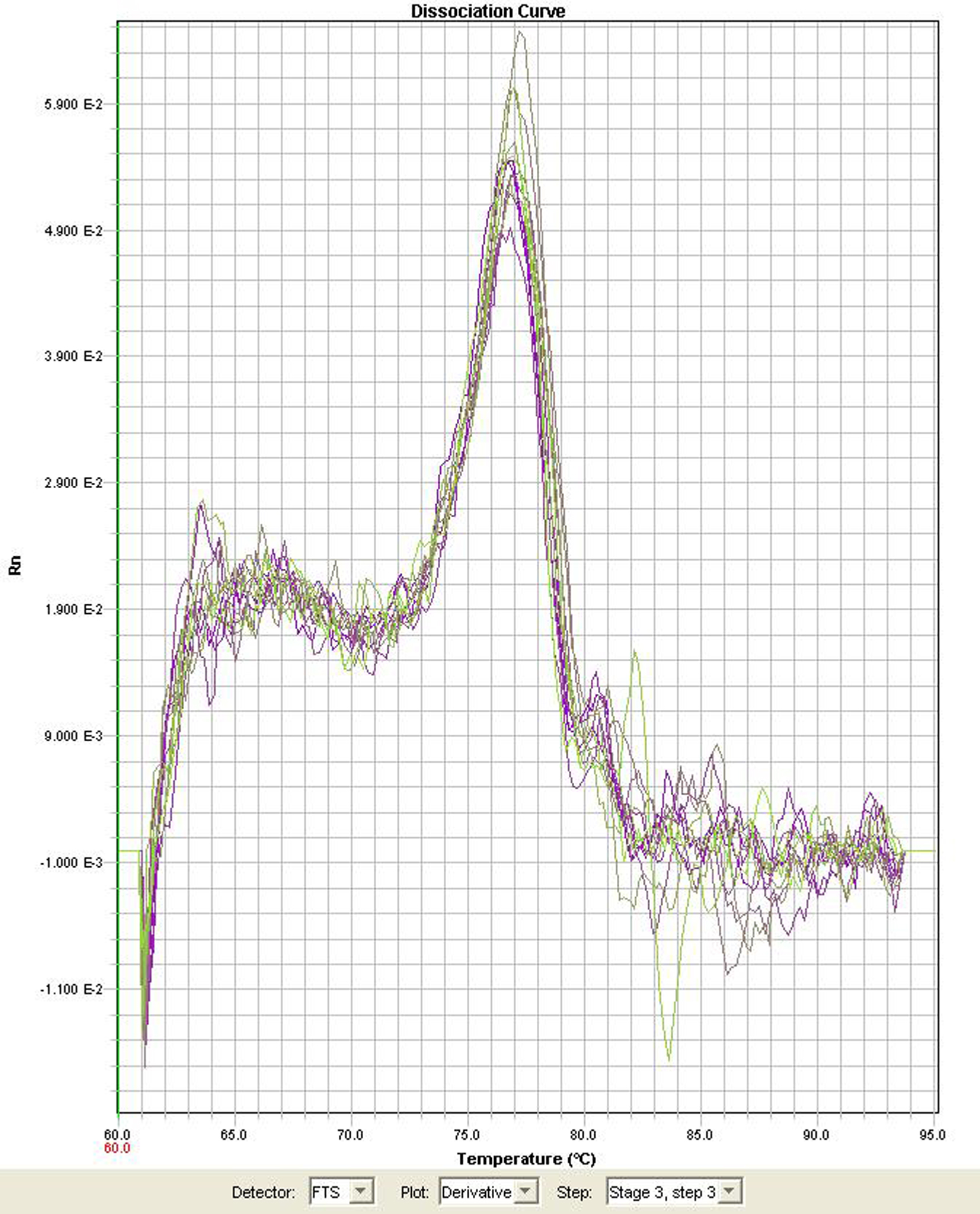

Supplement: File S1 — Contains: Figure S1. Effect of sub-MIC levels of Quercitrin (Q), Deoxynojirimycin (D) and the combinational effect of these compounds (Q+D) on enzymes associated with the acidurity and acidogenicity ofS. mutans. (a) F- ATPase activity (b) LDH activity and (c) Enolase activity. The significant difference compared with the control (P<0.05). Data represent mean± SD (n = 3). Figure S2. The dissociation curves of endogenous controlGyrA and FtsZare shown in panel (a), (b) and (c) respectively. (DOC) [file pone.0091736.s001.doc]
